# Preoperative cholesterol level as a new independent predictive factor of survival in patients with metastatic renal cell carcinoma treated with cyto-reductive nephrectomy

**DOI:** 10.1186/s12885-017-3322-5

**Published:** 2017-05-25

**Authors:** Hakmin Lee, Yong June Kim, Eu Chang Hwang, Seok Ho Kang, Sung-Hoo Hong, Jinsoo Chung, Tae Gyun Kwon, Cheol Kwak, Hyeon Hoe Kim, Jong Jin Oh, Sang Chul Lee, Sung Kyu Hong, Sang Eun Lee, Seok-Soo Byun, KOrean Renal Cell Carcinoma (KORCC) Group

**Affiliations:** 1 Department of Urology, Seoul National University Bundang Hospital, Seongnam, South Korea; 2 Department of Urology, Chungbuk National University College of Medicine, Cheongju, South Korea; 3 Department of Urology, Chonnam National University Hwasun Hospital, Hwasun, South Korea; 4 Department of Urology, Korea University School of Medicine, Seoul, South Korea; 5 Department of Urology, College of Medicine, The Catholic University of Korea, Seoul, South Korea; 6 Department of Urology, National Cancer Center, Goyang, South Korea; 7 Department of Urology, Kyungpook National University College of Medicine, Daegu, South Korea; 8 Department of Urology, Seoul National University Hospital, Seoul, Republic of Korea; 9 Department of Urology, Seoul National University College of Medicine, Seoul, South Korea; 10 Seoul National University Bundang Hospital, Seongnam, South Korea

**Keywords:** Renal cell carcinoma, Cholesterol, Survival, Metastasis, Hypercholesterolemia

## Abstract

**Background:**

The obesity and lipid metabolism were previously proposed to be related with the clinical outcomes of metastatic renal cell carcinoma (mRCC). We tried to investigate the relationship between preoperative cholesterol level (PCL) and survival outcomes in patients with mRCC.

**Methods:**

We analysed the data of 244 patients initially treated with cyto-reductive nephrectomy after being diagnosed with mRCC. Patients were stratified into two groups according to the PCL cut-off level of 170 mg/dL. The postoperative survival rates were compared using Kaplan-Meier analysis and the possible predictors of patients’ cancer-specific survival (CSS) and overall survival (OS) were tested using multivariate Cox-proportional hazard models.

**Results:**

The low cholesterol group showed significantly worse postoperative CSS (*p* = 0.013) and OS (*p* = 0.009) than the high cholesterol group. On multivariate analysis, low PCL was revealed as an independent predictor of worse CSS (hazard ratio [HR], 2.162; 95% CI, 1.221–3.829; *p* = 0.008) and OS (HR, 2.013; 95% CI, 1.206–3.361; *p* = 0.007). Subsequent subgroup analysis showed that these results were maintained in the clear cell subgroup but not in the non-clear cell subgroup.

**Conclusion:**

Decreased PCL was significantly correlated with worse survival outcomes in patients with mRCC treated with cytoreductive nephrectomy. The underlined mechanism is still uncharted and requires further investigation.

## Background

Renal cell carcinoma (RCC) is the most frequently diagnosed renal malignancy [1]. Owing to the constant advances of modern imaging technologies, the percentage of incidentally detected renal tumours has constantly increased during the last couple of decades [2, 3]. Although those phenomena brought the overall stage downward migration, a good percentage of patients are still diagnosed with metastatic renal cell carcinoma (mRCC) [3]. The use of cytoreductive nephrectomy in these patients with mRCC was reported to have significant survival benefits in several studies [4]. Therefore, further understanding of prognostic biomarkers is becoming more clinically important in selecting adequate candidates for adjuvant or neoadjuvant therapies for patients with mRCC perioperatively.

Several studies have reported a significant inverse relationship between obesity and RCC prognosis [5–7]. Although obesity is a well-known risk factor for the development of RCC [7], most studies reported that obese patients show more favourable pathology and survival outcomes, a phenomenon known as the “obesity paradox” [5, 6]. A large multicentre study recently analysed a large multi-institutional database of patients with mRCC and showed that patients with a low body mass index (BMI) showed significant worse survival compared to those with a high BMI [6]. They also showed that the high fatty acid synthase (FAS) expression was observed in patients with low BMI was connected to the worse survival outcomes. Their results suggest that the lipid metabolism is one of the important tumour metabolic mechanisms that are essential to tumour survival and progression. Since cholesterol is an essential cellular component that plays a crucial role in lipid metabolism, preoperative serum cholesterol level (PCL) may have significant correlation with prognosis in RCC patients [8]. Unfortunately, only two studies investigated this subject, both of which included small samples of patients with localized RCC but none with mRCC. Therefore, here we aimed to investigate the possible associations of PLC with survival outcomes in patients with mRCC after cytoreductive nephrectomy.

## Methods

We retrospectively analysed the data of 281 patients diagnosed with mRCC and initially treated with nephrectomy at multiple centres of South Korea. The informed consent has been waived by an approval of our institutional ethical review boards due to retrospective design (IRB number: B-1702/384–102). After the exclusion of 37 patients (neoadjuvant therapy [*n* = 7], other malignancy [*n* = 13], incomplete information [*n* = 17]), we finally included 244 patients. The clinical and pathological information was retrieved from prospectively managed databases of each institution. Every patient was initially evaluated using chest computed tomography (CT) (or simple radiography), abdominal CT, and bone scan. The PCL was included in the routine chemistry panels which was performed as a part of preoperative anesthetic risk evaluation within 4 weeks preceding the surgery. If there were multiple measurements before the surgical treatment, mean values were regarded as representative.

**Fig. 1:**
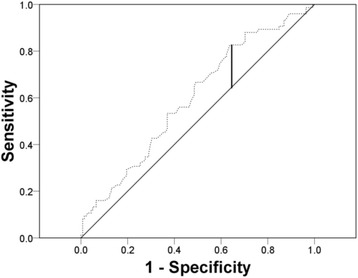
The receiver operating curve of preoperative cholesterol level upon cancer-specific mortality (*Vertical black line* indicates the points with maximal Youden’s value)

Pathological stage and histological subtype were determined according to the seventh TNM classification from the American Joint Committee Cancer Guidelines and the Heidelberg recommendations [9, 10]. The nuclear grades of the tumour cells were evaluated according to Fuhrman’s grading system [11]. The survival data and cause of death were determined by a rigorous review of the Korean National Statistical Office’s database and medical records of each hospital. The follow-up protocols varied slightly among institutions or physicians but usually included 3 month intervals after surgery. The receiver operating curve of PCL on the cancer-specific mortality was analysed and the area under the curve was 0.598. Since a PCL of 170 mg/dL showed the maximal Youden index value, the cut-off value was set at 170 mg/dL (Fig. 1). Therefore, the subjects with values ≥170 mg/dL were regarded the high PCL group and the others (PCL < 170 mg/dL) were regarded the low PCL group.

Independent T and chi-square tests were performed to compare the clinicopathological characteristics of the high and low PCL groups. To compare the survival outcomes of the two subgroups, Kaplan–Meier analyses were performed. Using multivariate Cox-proportional hazard models, the possible predictors of overall survival (OS), and cancer-specific survival (CSS) were tested. All of the statistical analyses were performed using SPSS software (version 19.0; SPSS, Chicago, IL, USA). All of the *p* values were two-sided and those <0.05 were considered statistically significant.

## Results

The clinical and pathological profiles of the entire cohort and subgroups according to the PCL are summarized in Table 1. The median age was 59.0 years (interquartile range [IQR], 52.0–68.0); median tumour diameter was 8.0 cm (IQR, 5.6–10.5), median PCL was 156.0 (IQR, 132.3–173.8), and median follow-up time was 13.0 months (IQR, 6.0–26.5). There were 88 patients in the high PCL group and 156 patients in the low PCL group. The low PCL group showed significantly lower haemoglobin level (*p* < 0.001) and higher platelet level (*p* = 0.038) than the high PCL group, but no significant differences were noted in the other clinical characteristics or pathological outcomes between the two groups.

**Table 1:** Summarization of clinico-pathologic factors of entire patients and according to the subgroups stratified by the cholesterol level of 170 mg/dL cut-off

	Entire patients (*n* = 244)	High PCL group (*n* = 72)	Low PCL group (*n* = 172)	*p* value
	Median (IQR) or Number (percent)			
Age (y)	59.0 (52.0–68.0)	57.5 (52.0–67.0)	60.0 (51.3–68.8)	0.981
BMI (*kg*/*m* ^2^)	23.0 (21.0–24.8)	23.5 (21.8–42.8)	22.9 (20.8–24.9)	0.239
Sex (male)	185 (75.8%)	50 (69.4%)	135 (78.5%)	0.091
Serum Albumin (g/dL)	4.0 (3.5–4.3)	4.3 (3.9–4.4)	3.9 (3.4–4.2)	< 0.001
Hemoglobin (g/dL)	12.1 (10.6–13.6)	13.1 (11.7–14.7)	11.8 (10.3–13.1)	< 0.001
Platelet (k/dL)	274.5 (132.3–173.8)	271.5 (212.5–320.5)	299.8 (222.0–378.8)	0.038
PLC (mg/dL)	156.0 (132.3–173.8)	190 (178–205.8)	139.1 (126.3–157.8)	
Corrected calcium (mg/dL)	9.2 (8.9–9.7)	9.4 (9.0–9.8)	9.5 (8.8–9.6)	
ECOG score (≥2)	79 (32.4%)	22 (30.6%)	57 (33.1%)	0.765
Diabetes mellitus	60 (24.7%)	14 (19.7%)	46 (26.7%)	0.326
Hypertension	120 (49.6%)	33 (46.5%)	87 (50.9%)	0.574
Tumor size (cm)	8.0 (5.6–10.5)	6.5 (5.0–9.5)	8.7 (6.1–11.0)	0.407
Clinical stage (≥3)	138 (56.6%)	32 (44.4%)	106 (61.6%)	0.059
Metastatic sites				0.541
Lung	78 (32.0%)	23 (31.9%)	55 (32.0%)	
Liver	7 (2.7%)	1 (1.4%)	5 (2.9%)	
Bone	24 (9.8%)	10 (13.9%)	14 (8.1%)	
Non-regional LNI	2 (1.0%)	0 (0%)	2 (1.2%)	
Adrenal gland	8 (3.3%)	4 (5.6%)	4 (2.3%)	
Multiple metastasis	10 (4.1%)	3 (4.2%)	7 (4.1%)	
Information missing	108 (44.3%)	33 (45.8%)	75 (43.6%)	
Miscellaneous	2 (1.0%)	0 (0%)	2 (1.2%)	
Pathologic stage				0.060
pT1	60 (24.6%)	24 (33.3%)	36 (20.9%)	
pT2	36 (14.8%)	11 (15.3%)	25 (14.5%)	
pT3	117 (48.0%)	33 (45.8%)	84 (48.8%)	
pT4	31 (12.7%)	4 (5.6%)	27 (15.7%)	
Fuhrman grade				0.424
≤ 2	34 (13.9%)	12 (16.7%)	22 (12.8%)	
≥ 3	210 (86.1%)	60 (83.3%)	150 (87.2%)	
Histologic subtype				0.214
Clear cell	213 (87.3%)	67 (93.1%)	146 (84.9%)	
Papillary	13 (5.3%)	2 (2.8%)	11 (6.4%)	
Chromophobe	4 (1.6%)	0 (0%)	4 (2.3%)	
Collecting duct	5 (2.0%)	1 (1.4%)	4 (2.3%)	
Unclassified	9 (3.7%)	2 (2.8%)	7 (4.1%)	

*IQR* interquartile range, *PCL* preoperative cholesterol level, *BMI* body mass index, *ECOG* Eastern Cooperative Oncology Group, *LNI* lymph node invasion

**Fig. 2:**
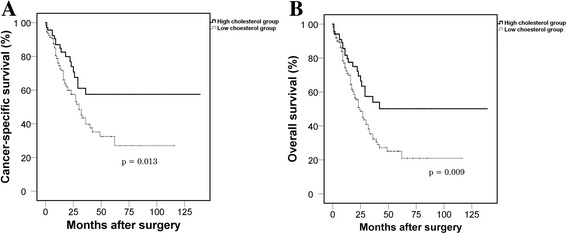
Kaplan-Meier analyses of cancer-specific survival (**a**) and overall survival (**b**) by preoperative cholesterol level

After a median follow-up of 12.0 months (IQR, 7.0–23.0), 85 patients died because of RCC. A total of 101 all-cause mortalities occurred after a median follow-up of 13.0 months (IQR, 7.0–23.5). The low PCL group showed significantly worse CSS (*p* = 0.013) and OS (*p* = 0.009) than the high PCL group (Fig. 2). The results from univariate Cox proportional analyses on CSS and OS were summarized in Table 2. Multivariate Cox proportional analysis revealed that low PCL was the independent predictor for worse CSS (HR, 2.162; 95% CI, 1.221–3.829; *p* = 0.008) and OS (HR, 2.013; 95% CI, 1.206–3.361; *p* = 0.007) (Table 3). When we stratified the patients by tumour histology (clear cell versus non-clear cell types), low PCL was revealed as an independent predictor for worse CSS (HR, 2.312; 95% CI, 1.274–4.193; *p* = 0.006) and OS (HR, 2.204; 95% CI, 1.279–3.799; *p* = 0.004) in the clear cell subgroup (Table 4). However, there were no significant relationships between PCL and survival outcomes in the non-clear cell subgroup (all *p* values >0.05). Subsequently, we further stratified the entire patient cohort into three risk groups (favourable, intermediate, poor) according to Heng’s model. We observed worse survival outcomes in the low PCL group, but the results did not reach statistical significance due to the small number of subjects (Table 4).

**Table 2:** Univariate Cox regression model adjusted for possible predictors estimating cancer-specific and overall survival in 244 patients treated with cyto-reductive nephrectomy for metastatic renal cell carcinoma

	Cancer-specific survival			Overall survival		
	HR	95% CI of HR	*p* value	HR	95% CI of HR	*p* value
Age	1.004	0.986–1.023	0.679	1.005	0.989–1.023	0.530
BMI (*kg*/*m* ^2^)	Reference (< 20)			Reference (< 20)		
20–25	0.778	0.456–1.327	0.356	0.783	0.478–1.283	0.332
≥ 25	0.373	0.182–0.762	0.007	0.387	0.202–0.743	0.004
Albumin (g/dL)	Reference (< 3.5)			Reference (< 3.5)		
3.5–4.3	0.610	0.375–0.991	0.046	0.577	0.371–0.899	0.015
≥ 4.3	0.515	0.290–0.916	0.024	0.485	0.286–0.822	0.007
Heng’s criteria	Reference (Low risk)			Reference (Low risk)		
Intermediate risk	1.265	0.729–2.198	0.403	1.188	0.720–1.961	0.499
High risk	1.842	0.957–3.546	0.067	1.806	0.999–3.266	0.050
Cholesterol level (cat.)	2.251	1.285–3.941	0.005	2.100	1.272–3.466	0.004

*HR* hazard ratio, *CI* confidence interval, *BMI* body mass index, *cat.* Categorical variable

**Table 3:** Multivariate Cox regression model adjusted for possible predictors estimating cancer-specific and overall survival in 244 patients treated with cyto-reductive nephrectomy for metastatic renal cell carcinoma

	Cancer-specific survival			Overall survival		
	HR	95% CI of HR	*p* value	HR	95% CI of HR	*p* value
Age	1.002	0.983–1.021	0.865	1.003	0.986–1.021	0.713
BMI (*kg*/*m* ^2^)	Reference (< 20)			Reference (< 20)		
20–25	0.792	0.459–1.369	0.404	0.805	0.485–1.336	0.402
≥ 25	0.443	0.202–0.924	0.030	0.466	0.232–0.936	0.032
Albumin (g/dL)	Reference (< 3.5)			Reference (< 3.5)		
3.5–4.3	0.776	0.440–1.370	0.382	0.716	0.426–1.204	0.208
≥ 4.3	0.784	0.399–1.544	0.482	0.713	0.383–1.328	0.287
Heng’s criteria	Reference (Low risk)			Reference (Low risk)		
Intermediate risk	1.095	0.623–1.926	0.752	1.033	0.619–1.725	0.902
High risk	1.185	0.550–2.553	0.664	1.132	0.565–2.270	0.727
Cholesterol level (cat.)	2.162	1.221–3.829	0.008	2.013	1.206–3.361	0.007
Age	1.002	0.983–1.021	0.830	1.003	0.986–1.021	0.701
BMI (*kg*/*m* ^2^)	Reference (< 20)			Reference (< 20)		
20–25	0.827	0.479–1.430	0.497	0.857	0.517–1.420	0.549
≥ 25	0.462	0.214–0.997	0.049	0.503	0.249–1.017	0.056
Albumin (g/dL)	Reference (< 3.5)			Reference (< 3.5)		
3.5–4.3	0.882	0.498–1.561	0.665	0.789	0.468–1.327	0.371
≥ 4.3	0.892	0.437–1.821	0.753	0.769	0.401–1.473	0.428
Heng’s criteria	Reference (Low risk)			Reference (Low risk)		
Intermediate	1.060	0.601–1.870	0.840	1.024	0.613–1.712	0.927
High risk	1.235	0.576–2.649	0.588	1.193	0.596–2.389	0.618
Cholesterol level (con.)	0.993	0.987–0.999	0.032	0.995	0.989–1.000	0.063

*HR* hazard ratio, *CI* confidence interval, *BMI* body mass index, *con.* Continuous variable, *cat.* Categorical variable

**Table 4:** Multivariate Cox hazard ratio models for the impact of low cholesterol on cancer-specific and overall survival after surgical treatment of metastatic renal cell carcinoma

	Cancer-specific survival			Overall survival		
	HR	95% CI of HR	*p*-value	HR	95% CI of HR	*p*-value
Entire cohorts	2.162	1.221–3.829	0.008	2.013	1.206–3.361	0.007
Subgroups according to the tumor histology						
Clear cell histology	2.312	1.274–4.193	0.006	2.204	1.279–3.799	0.004
Other histology	0.767	0.076–7.771	0.822	0.285	0.043–1.879	0.192
Subgroups according to the Heng’s model						
Favorable risk	1.556	0.487–4.967	0.455	1.767	0.602–5.185	0.300
Intermediate	1.809	0.907–3.611	0.093	1.583	0.851–2.947	0.147
Poor	1.538	0.339–6.972	0.577	1.735	0.406–7.416	0.457

Multivariate analyses were adjusted for age, body mass index, Heng’s risk group, preoperative albumin and cholesterol level. *HR* hazard ratio, *CI* confidence interval

## Discussion

In the present study, we found that low PCL was independently correlated with worse survival outcomes in mRCC patients treated by cytoreductive nephrectomy. Interestingly, PCL showed significant results in the clear cell type RCC but not in the non-clear cell RCC, which implies that lipid metabolism is mainly associated with clear cell subtype RCC. The PCL showed high HR in all three risk groups according to Heng’s criteria, although the results were non-significant due to the small number of included subjects.

Malignant cells have the notable feature of invasiveness and relentless proliferation, both of which require profound energy and raw materials. To support those abilities, most cancer cells have special metabolisms that enable them to promote their survival. This phenomenon has been termed “metabolic transformation” [12]. Among those, the most well-known metabolism in cancer cells is the “Warburg effect” [13]. Warburg et al. found that cancer cells produced adenosine triphosphate by non-aerobic glycolysis even in circumstances of sufficient oxygen, and this peculiar metabolism is beneficial because it produces less reactive oxygen species, which are hazardous to cancer cells due to the oxidative stress. Along with glucose metabolism, lipid metabolism is crucial to maintaining cancer proliferation and finishing the new building blocks because proliferating cells require plenty of nucleotides, fatty acids, membrane lipids, and proteins. Many cancer cells show high rates of de novo lipid synthesis [14].

Since cholesterol is an essential component of cellular membranes and important in energy production of tumour survival, the several previous studies investigated the relationship between cholesterol level and cancer development [15–17]. A large epidemiologic study analysed 33,368 Japanese subjects and concluded the presence of an increased incidence of stomach and liver cancers in patients having low cholesterol levels [15]. Another prospective study by Asano et al. also demonstrated that there were inverse association between cholesterol level and gastric cancer incidence after analysing the data of 2604 subjects for 14 years follow-up [16]. Kitahara et al. recently performed a retrospective analysis of a large database from South Korea with 1 million subjects and concluded that cholesterol level was correlated with increased incidence of several malignancies [17]. However, the influence of cholesterol was quite heterogeneous between the different malignancies. From their results, prostate, colon, and breast cancer showed high incidences in patients with high cholesterol, whereas liver, stomach, and lung cancer showed high incidences in patients with low cholesterol, showing that the relationship is quite variable and cancer-specific. Apart from the increased incidences, little has been investigated about the relationship between cholesterol level and cancer prognosis. Ohno et al. analysed 364 clear cell RCC patients and reported that a high PCL was associated with better CSS, although the findings of their multivariate analysis were not statistically significant due to a small number of subjects [18]. Another study by Martino et al. analysed a larger cohort of 867 subjects with localized RCC and concluded that low PCL independently correlated with worse CSS [19]. To our best knowledge, our study is the first to evaluate the prognostic value of PCL in patients with mRCC.

As the terminology “clear cell” indicates, the clear cell type of RCC accumulates significant amounts of cholesterol ester and glycogen in the cytosol [20]. Furthermore, several genes involved in lipid metabolism were previously reported to be related with clear cell type RCC progression [21]. In the present study, PCL showed significant associations in clear cell subtypes but not in non-clear cell subtypes, which implicates these relationship is intact only in the clear cell subtype. However, the exact mechanism or pathways underneath these phenomena are obscure and require elucidation.

Our study has several important limitations. First, the retrospective design and information gathering method are not immune to recall bias. Second, we could not analyse the influence of specific drugs such as statins. Third, patients received different salvage or palliative therapies from different attending physicians. Finally, we included only mRCC patients treated with nephrectomy, and further studies are needed to confirm our findings in all patients with mRCC.

## Conclusion

Preoperative serum cholesterol level was associated with worse survival outcomes in patients with mRCC after treatment with cytoreductive nephrectomy. Further basic studies are needed to elucidate the exact lipid metabolism underlying this peculiar phenomenon.

## Abbreviations

BMI: Body mass index
CSS: Cancer-specific survival;
CT: Computed tomography
FAS: Fatty acid synthase
HR: Hazard ratio
IQR: Interquartile range
mRCC: Metastatic renal cell carcinoma
OS: Overall survival
PCL: Preoperative serum cholesterol level
RCC: Renal cell carcinoma
